# Supplementary material for: Seafloor observations indicate spatial separation of coseismic and postseismic slips in the 2011 Tohoku earthquake
Source: Nat Commun. 2016 Nov 17;7:13506. doi: 10.1038/ncomms13506 (PMC5118554; doi:10.1038/ncomms13506)
Supplement: Supplementary Information — Supplementary Figures 1-4 [file ncomms13506-s1.pdf]

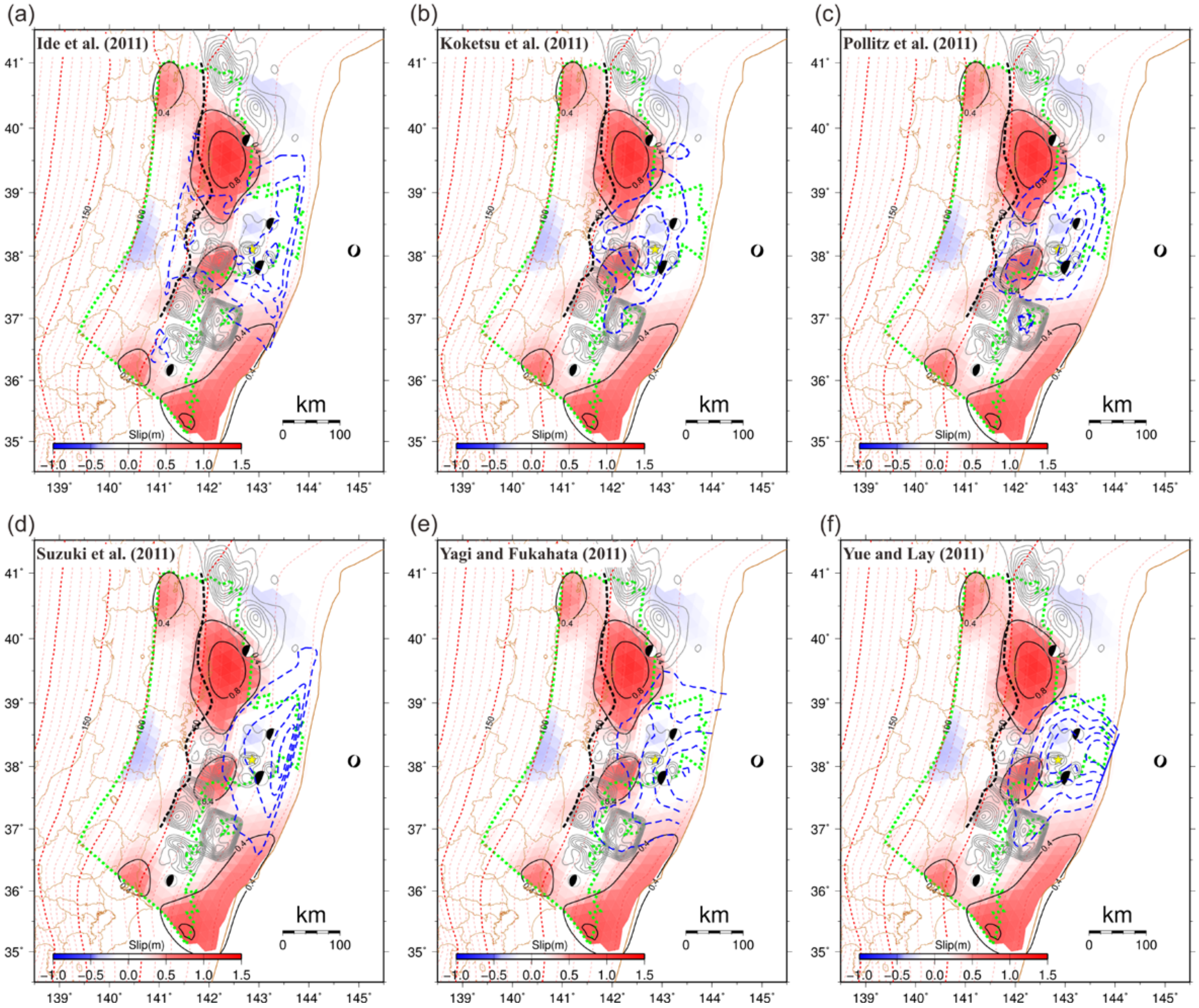

Supplementary Figure 1. Comparison with the various coseismic slip models. Comparison of estimated postseismic slip distribution (same as Figure 2(a)) and coseismic slip distributions of the 2011 Tohoku Earthquake. Coseismic slip distributions are from (a) Ide et al. (2011), (b) Koketsu et al. (2011), (c) Pollitz et al. (2011), (d) Suzuki et al. (2011), (e) Yagi and Fukahata (2011), and (f) Yue and Lay (2011). All contours for coseismic slip distributions are shown at 10-m intervals.

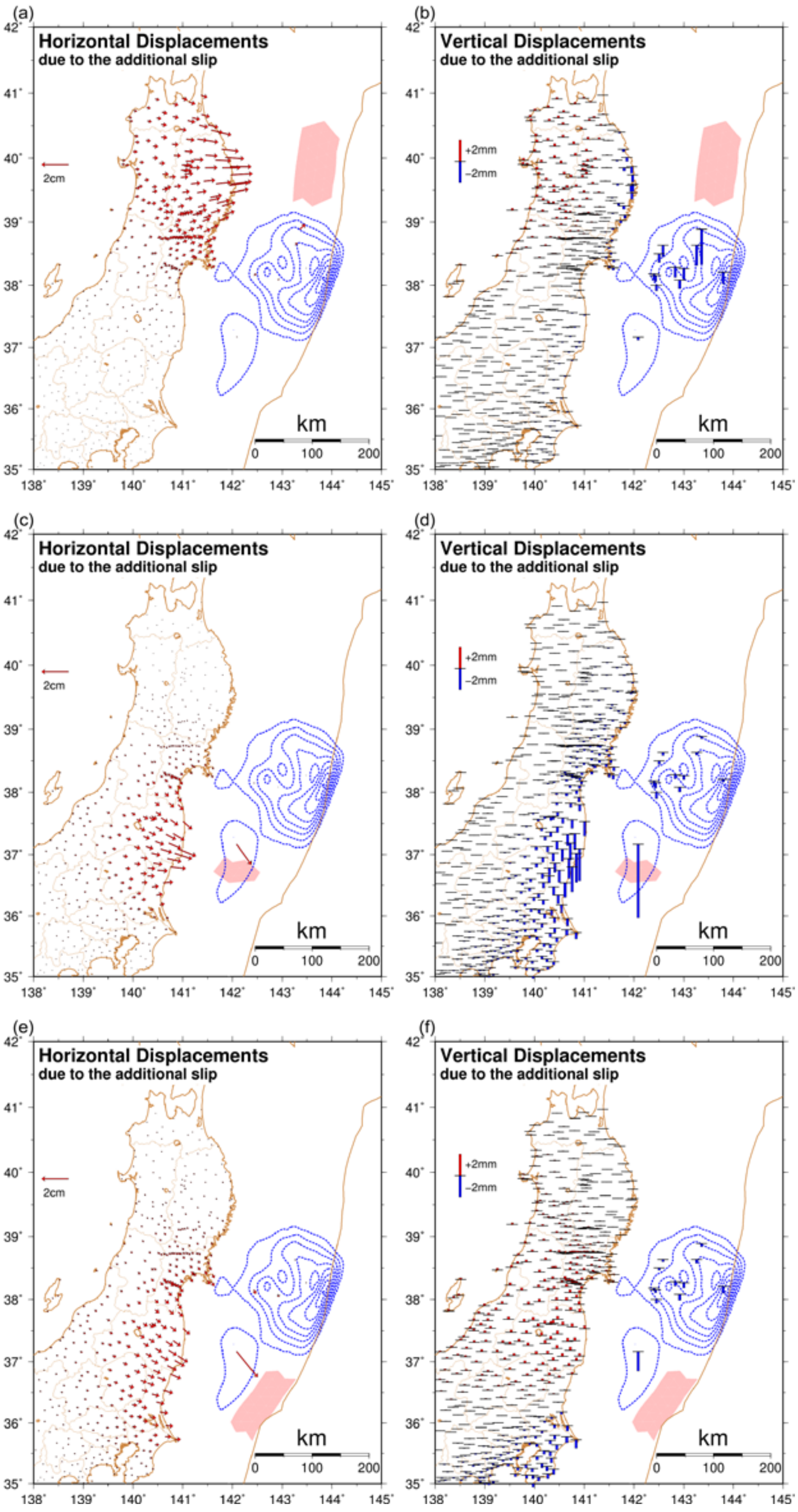

Supplementary Figure 2. Results of the examination about the detectability based on geodetic data. Calculated displacements due to the assumed slip on the plate interface. Reverse faulting slips are given to the triangular faults indicated by colour according to the given slip amounts with the same colour scale as in Figure. 1: (a) horizontal and (b) vertical displacements due to the slip far off Iwate Prefecture, (c) horizontal and (d) vertical displacements due to the slip off Fukushima Prefecture, and (e) horizontal and (f) vertical displacements due to the slip off Fukushima and Ibaraki Prefecture along the Japan Trench.

(a)

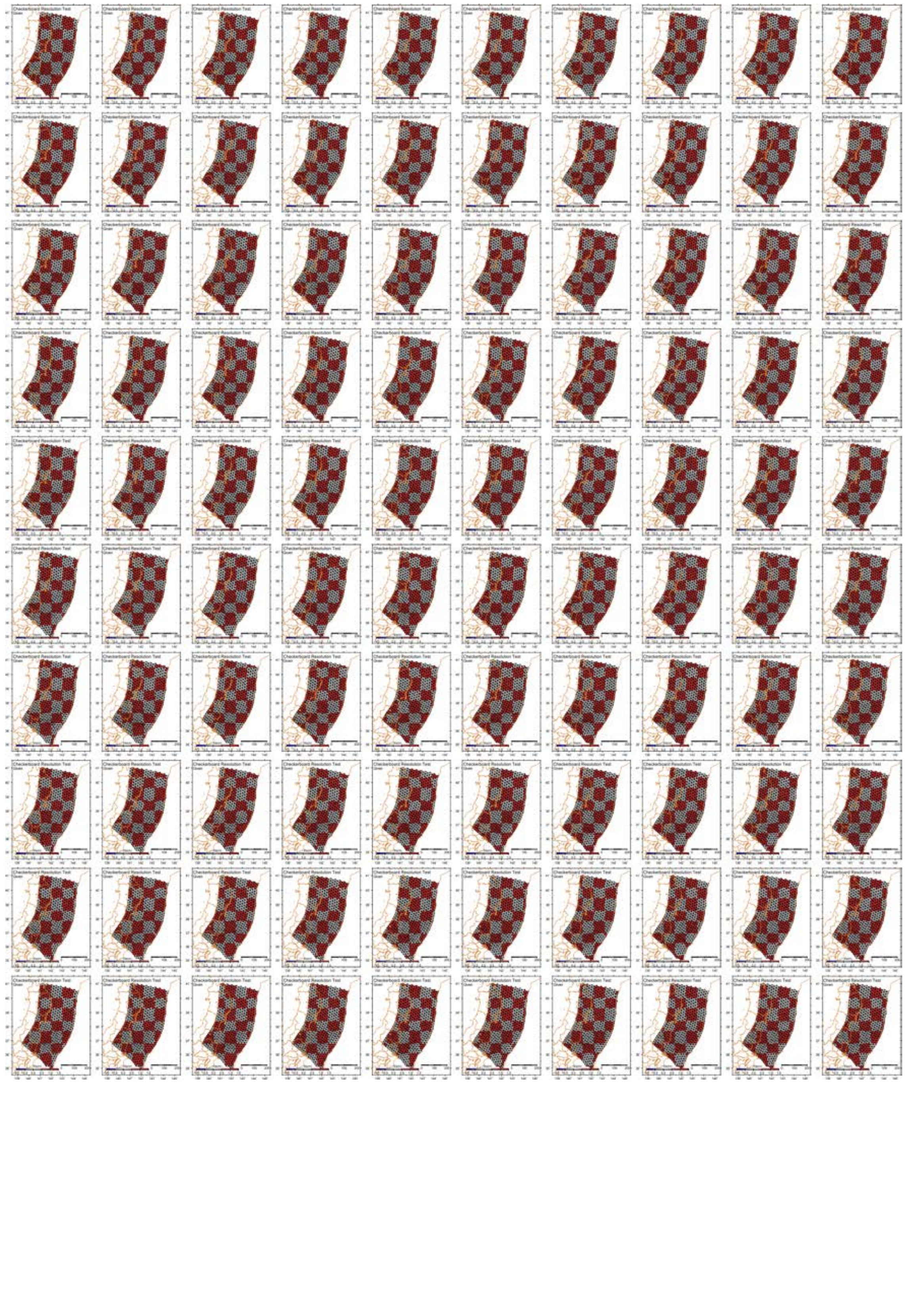

(b)

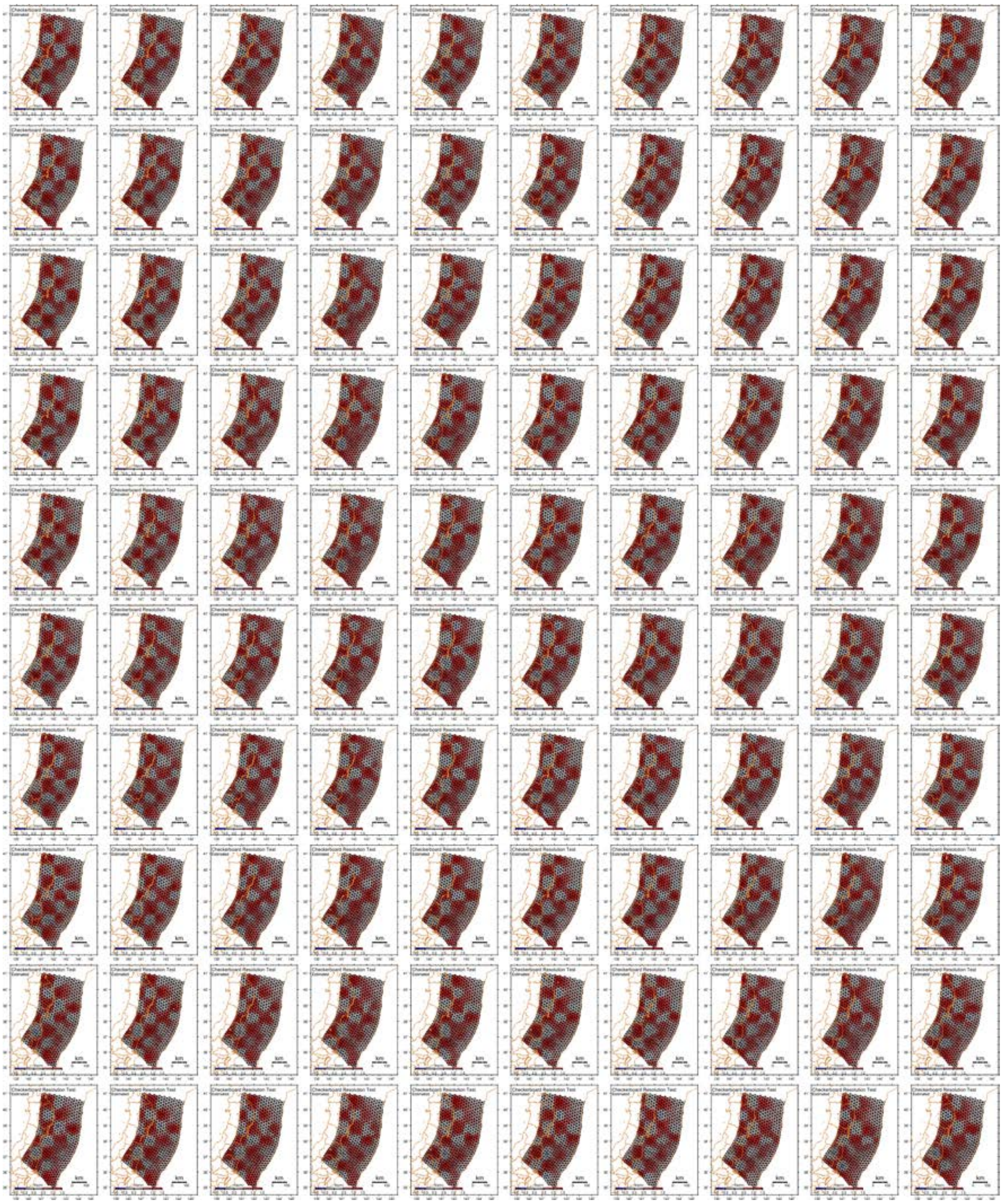

Supplementary Figure 3. Results of Checkerboard Resolution Tests (CRTs). Estimated slip distributions from the displacement fields synthesized based on the checkerboard patterns composed of patches with 1 and 0 m slips are shown. (a) The given slip distributions, in which the patch sizes are approximately 80 km parallel and perpendicular to the Japan Trench. The borders of the patches are changed both in N-S and E-W directions with 0.1° intervals. (b) Estimated slip distributions from the synthesized displacement fields. Rows and columns correspond to those in (a).

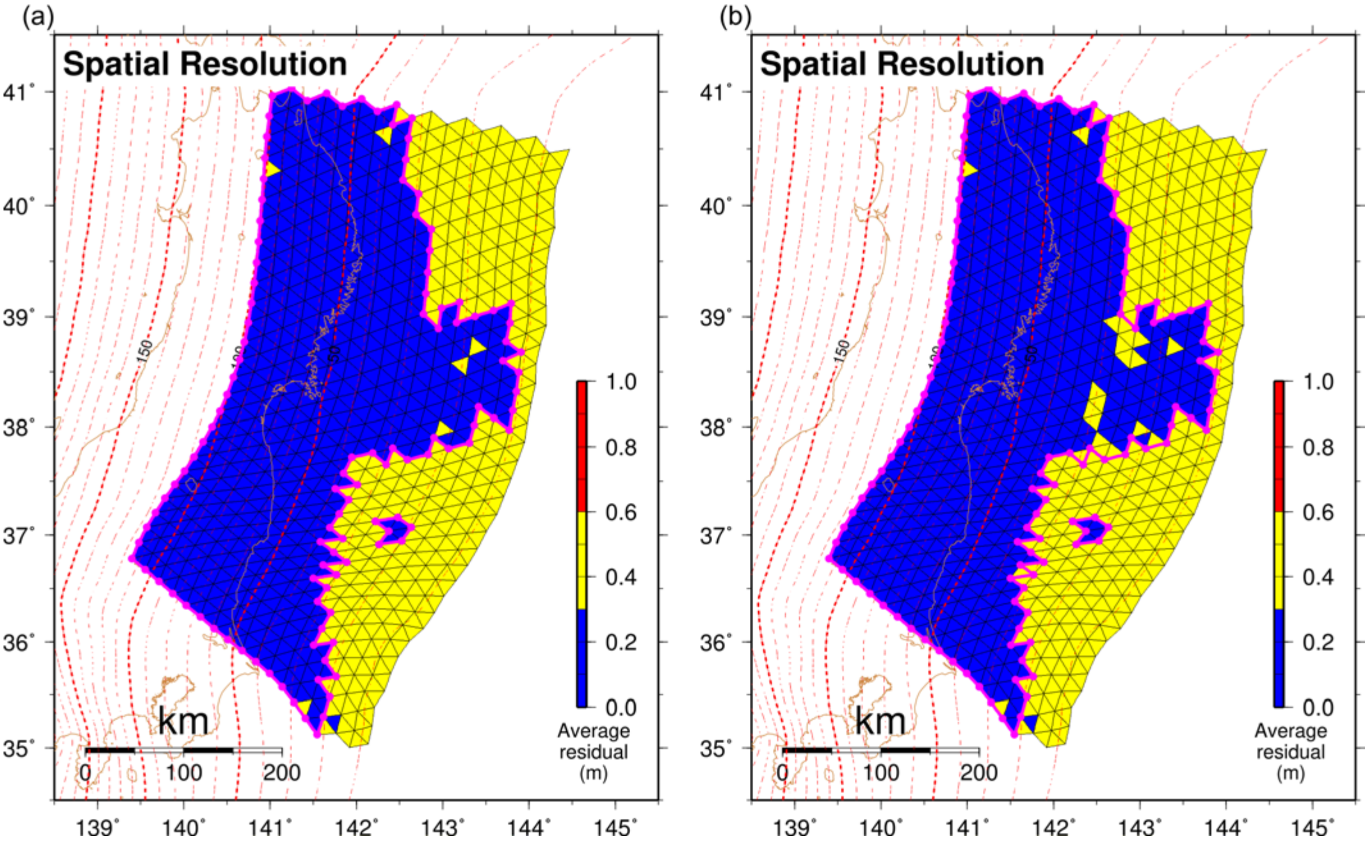

Supplementary Figure 4. Compiled results of CRT tests. (a) Spatial variation of the averaged residual between the given and estimated slips for the 100 cases of the checkerboard resolution test shown in Supplementary Figure 3. (b) Same as (a), but for the cases in which OBP data are excluded from the input to the slip inversion distributions.
